# Retrograde Flow to Aortic Root Predicts Inferior Cardiopulmonary Performance and Restrictive Lung Physiology in Fontan Circulation

**DOI:** 10.1016/j.cjcpc.2024.08.002

**Published:** 2024-08-23

**Authors:** Henri Juhani Pyykkönen, Otto Rahkonen, Heikki Tikkanen, Karim Khanji, Päivi Piirilä, Olli Pitkänen-Argillander

**Affiliations:** a Faculty of Health Sciences, School of Medicine, University of Eastern Finland, Kuopio, Finland; b Department of Pediatric Cardiology, New Children’s Hospital, Helsinki, Finland; c Unit of Clinical Physiology of the HUS Medical Diagnostic Center, Helsinki University Central Hospital and University of Helsinki, Helsinki, Finland

## Abstract

**Background:**

Cardiac output in Fontan circulation depends on systemic venous pressure, pulmonary vascular resistance, and ventricular function. Because myocardial function is dependent on coronary perfusion, we studied whether retrograde flow to aortic root in the mitral/aortic atresia subgroup of hypoplastic left heart syndrome (HLHS) affects cardiopulmonary performance.

**Methods:**

We studied 26 stable Fontan patients (14.4 ± 2.4 years) with right (RV, n = 17) and left (LV, n = 9) systemic ventricle morphology. All RV patients had HLHS and were subdivided according to postnatal flow to the hypoplastic ascending aorta being antegrade (HLHS-A) or retrograde (HLHS-R) due to valve atresia. Physical activity was assessed by questionnaire (LASERI, a questionnaire for Finnish children regarding physical activity), cardiopulmonary exercise test (1-minute ramp protocol), body composition (Biacorpus RX 4000), and muscle fitness (EUROFIT). These data were correlated with the postnatal aorta size and current branch pulmonary artery size index (McGoon index).

**Results:**

Patients with HLHS-R seldom self-reported engagement in vigorous physical activity and had significantly lower cardiopulmonary performance (peak oxygen uptake [VO_2_peak]) than patients with LV morphology (*P* = 0.037), but not compared with patients with HLHS-A. Branch pulmonary artery size did not correlate with VO_2_peak. Patients with HLHS-R had most severe lung restrictions (forced vital capacity *z*-score –3 ± 0.9, *P* = 0.0073; forced expiratory volume in 1 second *z*-score –3.3 ± 1.1, *P* = 0.001).

**Conclusions:**

Young Fontan patients with LV had better cardiopulmonary performance than patients with HLHS. Patients with HLHS-R were the least active and had the lowest VO_2_peak and most restrictive lungs. It is important to recognize postnatally single ventricle patients at high risk for inactivity to promote an active and healthy lifestyle.

## Résumé

**Contexte:**

En cas de circulation de Fontan, le débit cardiaque dépend de la pression veineuse systémique, de la résistance vasculaire pulmonaire et de la fonction ventriculaire. Puisque la fonction myocardique dépend de la perfusion coronarienne, nous avons évalué si le flux rétrograde vers la racine aortique avait une incidence sur la performance cardiopulmonaire (PCP) dans un sous-groupe de sujets présentant une atrésie aortique/mitrale en présence d’un syndrome d’hypoplasie du cœur gauche (SHCG).

**Méthodologie:**

Nous avons étudié 26 patients présentant une circulation de Fontan (14,4 ± 2,4 ans) avec morphologie ventriculaire systémique droite (VD, n = 17) et gauche (VG, n = 9). Les patients VD présentaient tous un SHCG et ont été répartis en sous-catégories selon que le flux vers l’aorte ascendante hypoplasique en postnatal était antérograde (SHCG-A) ou rétrograde (SHCG-R) en raison de l’atrésie valvulaire. L’activité physique a été évaluée à l’aide d’un questionnaire (LASERI, un questionnaire destiné aux enfants finlandais concernant l'activité physique), d’une épreuve d’effort cardiopulmonaire (protocole en rampe de 1 minute), la composition corporelle (Biacorpus rx4000) et la condition physique des muscles (EUROFIT). Ces données ont été mises en corrélation avec la taille de l’aorte en post-natal et l’indice basé sur le diamètre actuel des branches artérielles pulmonaires (indice de McGoon).

**Résultats:**

Les patients présentant un SHCG-R ont rarement indiqué avoir entrepris une activité physique intense et ont obtenu une performance cardiopulmonaire (absorption maximale d’oxygène ou VO_2_max) nettement moindre que les patients avec morphologie VG (*p* = 0,037), mais pas comparativement aux patients présentant un SHCG-A. Aucune corrélation n’a été établie entre le diamètre des branches artérielles pulmonaires et la VO_2_max. Les patients présentant un SHCG-R ont présenté les restrictions pulmonaires les plus sévères (score z pour la capacité vitale forcée : -3 ± 0,9, *p* = 0,0073; score z pour le volume expiratoire maximal en 1 seconde : -3,3 ± 1,1, *p* = 0,001).

**Conclusions:**

Les jeunes patients présentant une circulation de Fontan avec morphologie VG ont affiché une meilleure performance cardiopulmonaire que ceux présentant un SHCG. Les patients présentant un SHCG-R ont été moins actifs et ont présenté la VO_2_max la plus faible et les plus grandes contraintes pulmonaires. Il est important d’identifier en post-natal les patients présentant une circulation univentriculaire à risque élevé d’inactivité afin de promouvoir un mode de vie sain et actif.

Hypoplastic left heart syndrome (HLHS) is the most common type of single ventricle malformation. Palliative surgery in HLHS necessitates harnessing of the morphologic right ventricle (RV) to support the systemic circulation. In this first-stage operation (Norwood), mixing of blood at the atrial level is secured, neoaortic arch is created from the pulmonary trunk using patch augmentation, and the hypoplastic ascending aorta is implanted into the neoaorta by the Damus-Kaye-Stansel anastomosis. The pulmonary circulation is reconstituted initially by a systemic to pulmonary shunt and later by the stepwise direction of all systemic venous blood within the caval veins directly to the pulmonary branch arteries.^1^

In the cases of HLHS due to aortic and/or mitral valve atresia, the ascending aorta remains severely hypoplastic and the retrograde coronary perfusion of blood from the neoaorta into the hypoplastic aortic root may alter coronary perfusion and affect myocardial function. Fontan patients have a higher myocardial perfusion rate at rest than normal individuals. At stress, however, impaired preload affects haemodynamic flow reserves, and the kinetic energy of the forward flow of the blood is dissipated by the anatomic characteristics of the single ventricle.^2^^,^^3^

Studies in patients with palliated HLHS and unphysiological RV-systemic ventricle have demonstrated more abundant myocardial perfusion defects at rest-stress testing than the univentricular patients with dominant left ventricle (LV).^4^ During the exercise stress test, ischemic ST-segment changes are common in patients with HLHS despite no macroscopic evidence of coronary artery disease, and the response to adenosine-induced coronary flow is attenuated especially in patients with aortic or mitral atresia.^5^ These findings were considered suggestive that the type of HLHS with retrograde coronary flow may lead to attenuated myocardial perfusion, and impaired microcirculation and tissue structure of the systemic RV.^6^

In this study on Fontan patients in a stable condition, we hypothesized that retrograde aortic root filling would predispose to inferior physical performance when compared with patients with HLHS whose aortic filling is antegrade, or patients with systemic LV morphology and intact native aorta. In addition, we studied whether the size of branch pulmonary arteries influences cardiopulmonary performance of Fontan patients with either RV or LV morphology of the systemic ventricle.

## Methods

### Study design

We recruited 31 stable Fontan patients for the exercise measurements from the Pediatric University clinics in Finland. Patients were 8-17 years old and born anatomically with both LV and RV morphology. All patients had coped with normal everyday life without any obvious end-organ injury. Exclusion criteria were neurologic impediment, short stature, disturbed motor skills, pacemaker therapy for atrioventricular conduction block, and failing Fontan. We excluded 5 patients from this patient group because they failed to meet the anatomic requirements.

The postnatal diameter and flow direction of the native aorta were determined by echocardiography within the first 48 hours after birth. The latest paediatric cardiac magnetic resonance imaging (MRI) from all patients performed 12 ± 2.6 years after Fontan completion was used to assess the diameter of the descending aorta (diaphragmatic level) and the sizes of the branch pulmonary arteries. To normalize the pulmonary artery size at the hilar level with growth, their diameters were expressed as *z*-scores^7^ and normalized to the aortic size at the diaphragmatic level. Because the direction of blood flow to the aortic root and coronary arteries is determined by patency of the aortic valve already from the fetal period, we additionally studied whether the size of the ascending aorta at birth would correlate with exercise performance.

All RV patients had HLHS and were subdivided according to postnatal flow to the hypoplastic aortic root being antegrade (HLHS-A) or retrograde due to valve atresia (HLHS-R). No patient had more than a mild atrioventricular valve regurgitation of the systemic ventricle without statistical significance between the groups. The anatomy of the heart defects is outlined in Table 1.

**Table 1:** Demographic and anatomic characteristics including data on body composition and the latest MRI imaging of the 26 study patients with Fontan circulation

Anthropometry	LV-SVM (n = 9)	HLHS-A (n = 8)	HLHS-R (n = 9)	*P*	η^2^
Age (y)	14.3 ± 2.8	14.3 ± 1.7	15.0 ± 3.0	0.807	0.018
Height (cm)	160.2 ± 16.1	164.3 ± 8.8	162.0 ± 13.0	0.820	0.017
Weight (kg)	52.2 ± 19.7	52.8 ± 13.4	52.6 ± 14.7	0.996	0.00002
BSA	1.60 ± 0.30	1.65 ± 0.32	1.50 ± 0.31	0.545	0.096
Gender (female/male)	3/6	2/6	4/5	0.724	0.027
FM (%)	16.7 ± 11.2	16.1 ± 7.7	22.9 ± 9.0	0.208	0.113
Body mass index (kg/m^2^)	19 ± 4	19 ± 3	20 ± 4	0.976	0.010
Heart defect	DILV (3), TGA (4), TA (2), RV-hypoplasia (2)	HLHS (8)	MA (1), MA/AoVa (5), HLHS (8), PAPVD (1)		
Lower leg body mass (kg/cm)	0.33	0.31	0.29	0.502	0.060
Age at Fontan (y)	3.1 ± 0.5	2.9 ± 0.5	3.0 ± 0.5	0.646	0.037
Residual fenestration	0	0	0		
Number of sternotomies	3.3 ± 0.7	3.4 ± 0.5	3.2 ± 0.4	0.111	0.17
Postnatal echocardiogram					
Ao-asc (mm)	8.8 ± 3.6	5.9 ± 1.8	2.8 ± 1.1	0.045	0.235
MRI					
Years after Fontan operation	11 ± 2.6	12.7 ± 1.4	12.18 ± 1.4	0.193	0.06
Ao-asc (mm)	26.9 ± 5.7	12.1 ± 5.3	7.1 ± 2	0.0001	0.913
EF (%)	56.6 ± 7	54.1 ± 10	58.2 ± 7	0.597	0.083
RPA (*z*)	0.8 ± 1.0	0.6 ± 1.2	–0.09 ± 1.4	0.676	0.104
LPA (*z*)	2.0 ± 6.5	0.4 ± 0.7	–0.5 ± 1.7	0.476	0.068
Aorta at diaphragm (*z*)	0.0 ± 1.2	0.4 ± 1.1	0.2 ± 2	0.673	0.0146
McGoon index	2.3 ± 0.4	2.0 ± 0.3	1.7 ± 0.4	0.011	0.33

Data are presented as mean ± 1 standard deviation. The *P* values and effect sizes (η^2^) for analysis of variance indicate the statistical significance and magnitude of differences between the groups. A *P* value of <0.05 is considered statistically significant.

Ao-asc, diameter of ascending aorta; AoVa, aortica valve atresia; BMI, body mass index; BSA, body surface area; DILV, double inlet left ventricle; EF, ejection fraction; FM, fat mass; HLHS-A, hypoplastic left heart syndrome-antegrade; HLHS-R, HLHS-retrograde; LPA, left pulmonary artery; LV, left ventricle; MA, mitral atresia; MRI, magnetic resonance imaging; PAPVD, partially anomalous pulmonary venous drainage; RPA, right pulmonary artery; RV, right ventricle; SVM, systemic ventricle morphology; TA, tricuspid atresia; TGA, transposition of the great arteries.

### Patient and public involvement

#### LASERI questionnaire

The initial interview and the measurements of stature defined data on the patients’ physical condition and amount of their daily exercise. All patients arrived at the test with their guardians and filled the questionnaire together. We applied the Finnish LASERI (a questionnaire for Finnish children regarding physical activity) study, which has been used to investigate the relations of children’s physical activity to the risk factors of coronary artery disease since the 1980s.^8^ Our key questions were as follows:

(1) Do you feel yourself healthy or almost healthy?
(2) I can participate in every sports activity that I want.
(3) How many minutes (min.) per week do you exercise to the point of being out of breath?
(4) Are you participating in any guided regular sports activity?
(5) Do you feel like you can keep the same running pace as your classmates or teammates?
(6) Do you get out of breath more easily during sport than your classmates or teammates?

In questions 1, 5, and 6, the rating scale was in 1-4, where number 1 indicates minimum score and number 4 maximum score. In question 3, patients self-evaluated their own exercise amount, and question 4 was yes or no question.

### Cardiopulmonary exercise testing

Cardiopulmonary exercise testing (CPET) was performed by using a cycle ergometer (Ergoselect 200P; ergoline GmbH, Bitz, Germany) with paediatric pedals. Ramp of resistance was determined and increased at 1-minute steps 15 W/1 min or 20 W/1 min according to the height of the individual. The exercise was continued until the respiratory exchange ratio was at least 1.0 and the subjective level 17-19/20 on the Borg scale for perceived exertion. Work rate was expressed as the peak work rate (W) as well as percentage of the predicted value according to Harkel et al.^9^ Arterial saturation (SaO_2_) was assessed noninvasively with oximeters: one on the fingertip and the other attached to the earlobe (MySign S oximeter; Envitec GmbH, Wismar, Germany).

**Figure 2:**
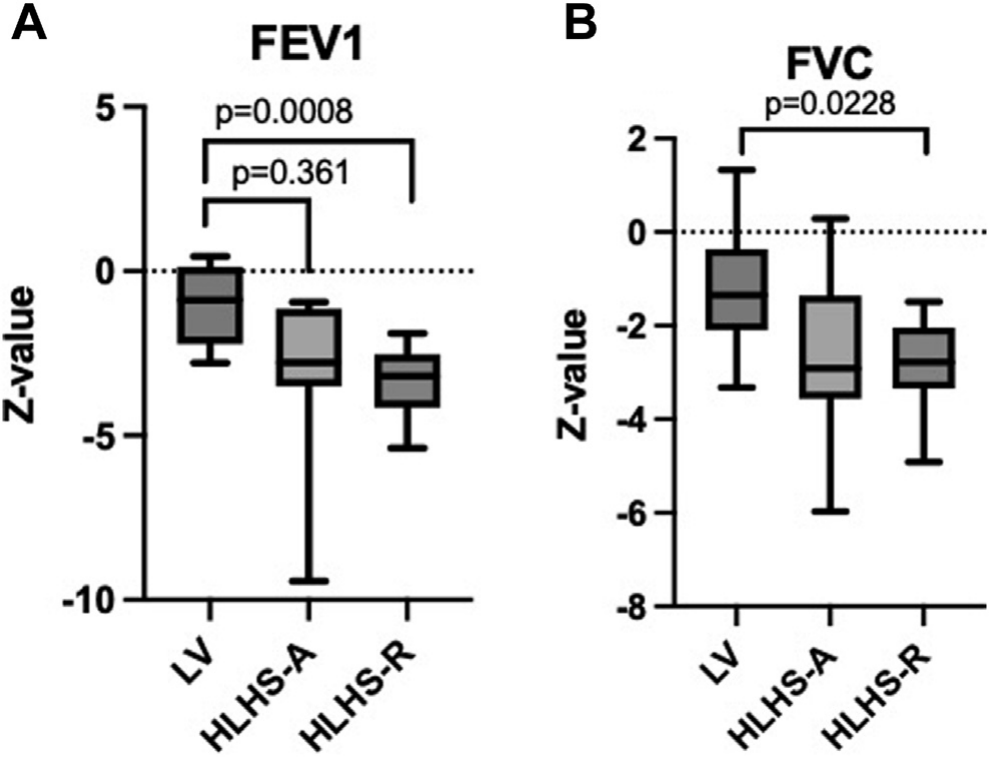
(**A**) In spirometry tests, LV patients had higher FEV_1_*z*-score than both HLHS groups (*P* = 0.0361 and *P* < 0.005). (**B**) LV group demonstrated significantly higher FVC *z*-scores in spirometry than patients with HLHS-R (*P* = 0.0228). FEV_1_, forced expiratory volume 1 second; FVC, forced vital capacity; HLHS-A, hypoplastic left heart syndrome-antegrade; HLHS-R, HLHS-retrograde; LV, left ventricle; RV, right ventricle.

Breath-by-breath gas analysis was performed using Vyntus CPET (CareFusion 234 GmbH, Hoechberg, Germany). For measurement of respiratory gases, a tightly attached face mask (Rudolph series 7910; Hans Rudolph, Kansas City, MI) was used, and the dead space values were added to the program. The ventilatory threshold 2 was assessed at the point of slope change of VCO_2_ exceeding VO_2_, increase of VE/VO_2_ compared with VE/VCO_2_, and increase of PetO_2_ vs PetCO_2_ (partial pressures of O_2_ and CO_2_ in expiratory air).^10^ Through the Vyntus CPET device, spirometry was performed before the test, and forced expiratory volume in 1 second was followed up immediately after the end of exercise and 4 and 10 minutes after exercise. Flow-volume loops were measured at every second step. The measured parameters are shown in Table 2 (see also Fig. 2).

**Table 2:** Data on cardiopulmonary exercise test of 26 paediatric patients with Fontan circulation and 2 different systemic ventricle morphologies

CPET results	LV-SVM (n = 9)	HLHS-A (n = 8)	HLHS-R (n = 9)	One-way ANOVA (*P*)
SpO_2_ (%)	94 ± 3	94 ± 4	93 ± 6	0.741
VO_2_peak (mL/kg/min)	32.5 ± 5.8∗	30.0 ± 4.2	25.4 ± 5.9∗	0.044 ∗0.0376
HR peak	170 ± 19	169 ± 17	169 ± 11	0.426
VO_2_/HR	9.7 ± 2.2	9.2 ± 1.8	7.8 ± 1.5	0.096
VE/VCO_2_	31.6 ± 7	32,4 ± 8	33,3 ± 6	0.874
VT2 (mL/kg/min)	21.5 ± 4.3	21.1 ± 4.3	18.6 ± 5.2	0.2795
BP peak (mm Hg)	174.7 ± 22.7	162.9 ± 23.4	165.5 ± 29.6	0.486
Peak workload (W)	133 ± 9	127 ± 26	104 ± 32	0.210
FVC (L)	3.3 ± 1.2	3.0 ± 0.9	2.7 ± 0.5	0.580
FVC (*z*)	–1.20 ± 1.3∗	–2.7 ± 1.9	–3.0 ± 0.9∗	0.044 ∗0.0073
FEV_1_ (L)	3.0 ± 1.0	2.6 ± 0.8	2.3 ± 0.6	0.237
FEV_1_ (*z*)	–1.0 ± 1.2∗	–3.2 ± 2.7	–3.3 ± 1.1∗	0.019 ∗0.028
FEV_1_/FVC (%)	92	83	85	0.843
FEV_1_/FVC (*z*)	0.6 ± 0.8	–1.0 ± 2.0	–0.7 ± 1.4	0.843

Data are presented as mean ± 1 standard deviation. The *P* values indicate the statistical significance from 1-way ANOVA tests. A *P* value of <0.05 is considered statistically significant.

ANOVA, analysis of variance; BP, blood pressure; CPET, cardiopulmonary exercise testing; FEV_1_, forced expiratory volume 1 second; FVC, forced vital capacity; HLHS-A, hypoplastic left heart syndrome antegrade aortic root filling; HLHS-R, hypoplastic left heart syndrome, retrograde aortic root filling; HR peak, peak heart rate; HSD, Honestly Significant Difference test; LV, left ventricle; SVM, systemic ventricle morphology; SpO_2_%, oxygen saturation; VO_2_peak, peak oxygen uptake; VT2, ventilatory threshold II.

∗ VO_2_peak and FEV_1_ (*z*) comparisons were analyzed for specific pairwise differences using Tukey’s HSD test. Additionally, pairwise comparisons for FVC (*z*) were also performed using the Mann-Whitney *U* test.

### Body composition

Bioelectrical impedance analyses were used to estimate patients’ fat percentage and fat-free muscle mass. The body bioelectrical impedance analyses measurement was made in a fasted state after a visit to the toilet. We used a Biacorpus RX 4000 analyzer suitable for paediatric patients exploiting a reference data pool included in its BodyComposition Version 9.0 Professional software (MEDI CAL HealthCare GmbH, Karlsruhe, Germany).^11^ For calculations, the combined muscle mass of the legs was indexed against the patient’s weight, both expressed as kilograms.

### Statistical analysis

Explanatory factors, which were continuous variables, were initially screened using multiple regression analysis. Group comparisons were made using 1-way analysis of variance (ANOVA), and pairwise comparisons were performed with *post hoc* tests (Tukey Honestly Significant Difference test) or the Mann-Whitney *U* test, as appropriate. Pearson’s correlation analysis was used to assess the statistical relationship between the size of the ascending aorta at birth and peak oxygen uptake (VO_2_peak). Effect sizes were calculated using Eta squared (η^2^) to evaluate the magnitude of differences observation in the 1-way ANOVA. Data are expressed as mean ± standard deviation; *P* < 0.05 was considered significant.

## Results

### Self-reported physical activity

According to the patient self-reported LASERI questionnaire, 55% of patients reported rarely feeling out of breath during exercise, whereas 45% felt more exhausted than their healthy counterparts. The majority of patients (70%) could not maintain the same running pace or duration as their healthy peers, regardless of ventricular morphology. Altogether 50% of patients in the LV group participated guided sports club activities in junior semicompetitive level. Participation rates were 40% and 20% in the HLHS-A and HLHS-R groups, respectively. The different sports that were represented included ice hockey (2), floorball (2), cheerleading (2), Finnish baseball (1), bowling (1), and gymnastics (1).

**Figure 1:**
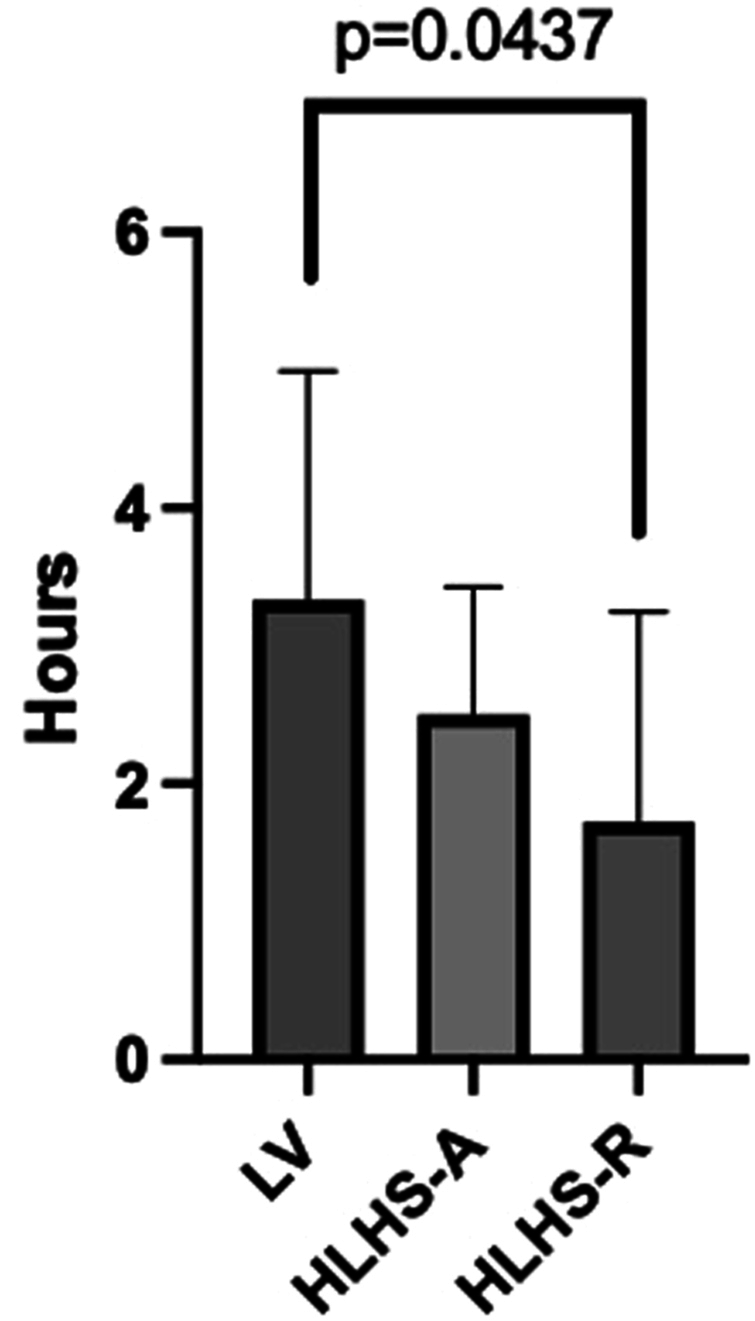
Weekly amount of vigorous exercise (hours) of 26 Fontan patients with LV as the systemic ventricle (n = 9) in comparison with patients with systemic RV and anterograde (HLHS-A, n = 9) or retrograde (HLHS-R, n = 8) flow to the native aortic root, *P* = 0.043 and η^2^ = 0.201 (Mann-Whitney *U* test, η^2^). HLHS-A, hypoplastic left heart syndrome-antegrade; HLHS-R, HLHS-retrograde; LV, left ventricle; RV, right ventricle.

LV patients reported engagement of vigorous exercise activity for a median 3.3 ± 1.6 hours per week, patients with HLHS-A 2.4 ± 1 hours, and the subgroup of HLHS-R engaged in brisk exercise only 1.7 ± 1.5 hours weekly. A statistical difference was observed between the LV and HLHS-R group, but not between the LV and HLHS-A group (*P* = 0.043) with an effect size of η^2^ = 0.201. The results of physical activity are shown in Figure 1.

### MRI results

The most recent MRI of the patients was used to measure sizes of branch pulmonary arteries (Table 1). Before calculating the McGoon index, the growth of the native aorta at the diaphragmatic level was confirmed to be similar between the groups. We found that the pulmonary branch artery size was greater in patients with LV as the systemic ventricle than in patients with HLHS-R (*P* = 0.0151) but was not between the LV and HLHS-A groups. Systemic ventricular ejection fraction did not differ between patients.

### Cardiopulmonary exercise test

All patients (N = 26) successfully underwent a CPET to exhaustion, reaching the target respiratory quotient without experiencing any adverse events. The results of the 1-way ANOVA demonstrated a significant difference between groups (*P* = 0.044). *Post hoc* tests indicated that patients with LV mor had a superior VO_2_peak to patients with HLHS-R (*P* = 0.0376). The effect size, as measured by η^2^, was calculated to be 0.24. At the submaximal level, ventilatory threshold 2 did not differ among patients. The patients with HLHS-R demonstrated the lowest peak workload in comparison with the patients with LV and HLHS-A. The results of the CPET are presented in Table 2.

The pulmonary function tests demonstrated that the forced expiratory volume in 1 second was significantly decreased in all the Fontan study groups (Figure 2 and Table 2). It was closest to normal in LV patients and significantly better than in the HLHS-R group (*P* = 0.019), *post hoc* (*P* = 0.028) and η^2^ = 0.510. Also forced vital capacity was below normal in the patients with LV as the systemic ventricle, but significantly decreased in patients with HLHS-R (*P* = 0.0073). Branch pulmonary artery size did not correlate with VO_2_peak right pulmonary artery (*P* = 0.375, *R*^2^ = 0.035) and left pulmonary artery (*P* = 0.182, *R*^2^ = 0.076). However, the size of the ascending aorta at birth significantly correlated with VO_2_peak (*P* = 0.0382, *R*^2^ = 0.2561).

## Discussion

The key observation of our study was that the cardiopulmonary performance was lowest in paediatric and adolescent Fontan patients with HLHS anatomy and retrograde perfusion into the aortic root (mitral and/or aortic atresia). The diameter of the native ascending aorta at birth was smallest in patients with HLHS and demonstrated a negative correlation with VO_2_peak in our patient cohort. Importantly, all our patients had restrictive lung physiology that was pronounced in HLHS with retrograde flow to the aortic root. Our Fontan patients’ peak performance measured at VO_2_peak 29.5 ± 6.2 mL/kg/min attained at CPET was at the same level as reported previously, and approximately 64% ± 14% of their healthy peers.^12^, ^13^, ^14^, ^15^

Performance of Fontan circulation is dependent on pulmonary vascular resistance, myocardial properties, single ventricle loading conditions, heart rate, and pulmonary function.^14^^,^^16^ Cardiac function may be affected during preservation at the heart surgeries and the morphology of the systemic chamber because some available data suggest that the systemic LV may adapt better to the univentricular stages than the systemic RV.^14^^,^^17^^,^^18^ The study by Durongpisitkul et al.^12^ in 59 Fontan patients aged 5-41 years demonstrated subnormal peak VO_2_ and attenuated stroke volume and heart rate response to exercise without an obvious effect by the systemic ventricle.

The impact of the systemic ventricle morphology on cardiopulmonary performance has been tested in adult patients with Fontan circulation.^12^^,^^19^^,^^20^ Dhauna et al.^19^ recently followed 135 young adults with Fontan haemodynamics for 3-4 years and observed no difference in performance between patients with LV or RV systemic pumping ventricle nor deterioration of peak heart rate, oxygen saturation, and peak oxygen consumption between patients with RV and LV systemic chamber. In contrast, another study on young adults with a median age of 26.3 years reported that peak VO₂ and peak oxygen pulse were reduced in patients with RV systemic ventricle compared with patients with LV morphology or common ventricle.^20^ The findings of the present study corroborate with these latter findings and demonstrated inferior cardiopulmonary performance in HLHS with retrograde filling of the aortic root. Because attenuated changes in the coronary flow reserves and myocardial microcirculation of Fontan patients have previously been described particularly in HLHS,^6^ it is possible that our findings are at least partly due to defective coronary flow. To our knowledge, the present study is the first to compare Fontan patients with retrograde aortic flow and patients with antegrade aortic flow.

The observation that all our patients had restrictive lungs corroborates with another recent observation and may importantly play a role for cardiopulmonary capacity.^21^ A recent clinical study included 14 young adults aged 6-19 years after establishment of Fontan circulation, of whom 6 had restrictive ventilatory findings. Of these adolescent Fontan patients, 8 agreed to begin a 3-month-long controlled respiratory training where theoretical education was provided, and the awareness of respiration was increased by controlled weekly breathing exercises. After intervention, the patients were able to significantly increase exercise endurance time, minute ventilation, peak respiratory rate, and peak oxygen consumption from 21.3 up to 27.8 mL/kg/min.^22^ Furthermore, the response of lung function to respiratory muscle weakness was recently studied in 23 adolescent Fontan patients. The 6-week training period resulted in improved ventilation observed as more vigorous inspiratory pressure development without affecting the expirium. However, training of the inspiratory muscles had no effect on peak VO₂, oxygen pulse, or the Borg score.^23^

The inherently low cardiopulmonary capacity of Fontan patients has been associated with reduced peripheral oxygen saturation, reduced vital capacity with increased dead-space ventilation, low vital capacity, and thoracic dysfunction possibly induced by multiple sternotomies.^12^ The paediatric patients in the present study had undergone similar amount of chest operations. Interestingly, we observed that patients with the smallest branch pulmonary artery sizes demonstrated the lowest lung volumes and forced expiratory volumes indicating lung restriction. These patients also had the smallest native ascending aorta size at birth and retrograde aortic root filling persisting after the Fontan completion. To our knowledge, this has not been observed previously.

The exercise behaviour of children and adolescents is typically influenced by internal motivation and guidance from other adults such as coaches, teachers, and peers.^24^ The current daily exercise recommendation for Finnish school children at 7-18 years of age is 1-2 hours of many-sided exercise suitable for age. There are some data on success rate on this that suggest that 33% of the kids at 7-15 years of age reach this aim, but at high school this drops to 10% or even less.^25^ In terms of these considerations, the daily activity of our patients seems comparable to or better than their healthy peers. According to the LASERI questionnaire, the present patients with LV morphology were more active in everyday life and voluntary exercise but also participated in sports club activities more often than the patients with HLHS-R. It is possible that exercise-induced ventilation and demands for myocardial perfusion induce discomfort in the latter patients with most restrictive lungs and aberrant coronary flow.^26^

Recently it has been suggested that a physically active and healthy lifestyle with regular exercise is safe and should be promoted in all patients with Fontan circulation.^27^ Exercise interventions have demonstrated that self-efficacy, walking distance, exercise capacity, ventilatory properties, and peak oxygen consumption can be increased in these patients.^28^, ^29^, ^30^ In addition, a proper tone and strength of the peripheral muscles may be an important aid for generating propulsive support to facilitate transpulmonary flow.^31^ Finally, changes in exercise capacity may have prognostic value in predicting the future need of frequent hospitalizations or death and transplantation.^15^ Our data corroborate with these observations and provide insight on how anatomic subgroup variations of the single ventricles predict exercise performance. Our data can aid in prescribing exercise and monitoring cardiopulmonary performance stability.

### Study limitations

All study patients were in stable clinical condition with similar age range and body composition among the subgroups. There were no differences in resting global systolic functions in terms of ejection fraction of the systemic ventricles measured by cardiac MRI. In addition, the numbers of operations on chest were similar, suggesting that the musculoskeletal properties of the upper trunk at CPET were identical. The patients had subnormal but similar VE/VCO_2_ response, indicating similar cardiopulmonary competence for CO_2_ removal during exercise. A *post hoc* power analysis indicated that our study had a power of approximately 56.4% to detect medium-sized effects (η^2^ = 0.201) at a significance level of 0.05. In terms of these patient characteristics, we feel that the comparisons of cardiopulmonary performance were appropriate. The current study was able to tease out relevant statistical differences between patients with LV- and RV-systemic ventricle morphology. However, probably due to a relatively small number of patients, only trends among the RV-systemic ventricle morphology groups were observed. These nonsignificant trends deserve further investigation in our future studies with larger patient groups.

## Conclusions

In this study, we demonstrated in stable paediatric Fontan patients that there are potential differences how ventricular morphology may associate with voluntary exercise habits, peak oxygen consumption, and lung function. Single ventricle patients with LV morphology of the systemic ventricle outmatch their peers with HLHS in a cardiopulmonary exercise test. In HLHS, retrograde flow to the minute native aortic root predisposes to inferior cardiopulmonary performance. In addition, the branch pulmonary artery size was smaller and lung restriction on spirometry most severely compromised in the latter patients. These data should be helpful for tailoring exercise prescription and monitoring its stability of achievement in patients with Fontan haemodynamics.
